# Consumption Trends and Eating Context of Lentils and Dried Peas in the United States: A Nationally Representative Study

**DOI:** 10.3390/nu16020277

**Published:** 2024-01-17

**Authors:** Namrata Sanjeevi, Pablo Monsivais

**Affiliations:** Department of Nutrition and Exercise Physiology, Elson S. Floyd College of Medicine, Washington State University, Spokane, WA 99202, USA; p.monsivais@wsu.edu

**Keywords:** lentils and dried peas, NHANES, consumption trends

## Abstract

Background: Incorporation of lentils and dried peas could form the basis of a nutrient-rich diet; yet, they are among the least-consumed legumes in the United States (US). The objective of this study was to examine the prevalence of lentils/dried peas consumption in the US over time and across socio-demographic groups, as well as to examine the eating context of these foods. Methods: Analyses included adults (aged 18 years or older) and children (aged 3–17 years) participating in National Health and Nutrition Examination Survey (NHANES) 2003–2004 through 2017–2018. Participants consuming lentils/dried peas on one or both of the 24-h dietary recalls were categorized as consumers. Results: Although an increasing time trend in prevalence of consumption was observed over the study period, prevalence of lentils/dried peas consumption was less than 7% in NHANES 2017–2018 in adults and children. Demographic differences were observed, such that a greater proportion of non-Hispanic Asians were classified as consumers. Lentils/dried peas were primarily obtained from grocery stores and supermarkets. Conclusions: Although there are signs of rising acceptance of dried peas and lentils, the low prevalence of lentils/dried peas consumption suggests that understanding barriers to consumption of these foods could further identify opportunities to improve their consumption.

## 1. Introduction

Unhealthy dietary intake is a leading risk factor in the United States (US), contributing to excess rates of deaths from CVD, diabetes, and some cancers [1]. Shifting to more plant-focused diets could improve nutrition and also reduce the environmental impact of diets [2], yet such healthy diets are often more costly to consumers [3]. The 2020–2025 Dietary Guidelines for Americans (DGAs) emphasizes plant-based and minimally-processed dietary patterns [4]. The DGAs cite a robust evidence base indicating that plant-rich and diverse dietary patterns like the Dietary Approaches to Stop Hypertension (DASH) [5] and Mediterranean food patterns [6] can form the basis of a satisfying and nutrient-rich diet. A consistent component of these healthy diet patterns is legumes. Legumes are among the lowest-cost sources of protein and fiber [7,8]. Further, legumes have been shown to provide the highest nutritional value per penny compared to other vegetables [9]. Legumes, including lentils and dried peas [10] are an excellent source of folate [11,12,13] and soluble fiber, especially resistant starch, which have important prebiotic functions [14,15]. Although lentils and dried peas are an abundant source of dietary protein and fiber [16], they are not consumed in adequate quantities for health [17] and are among the least-consumed legumes in the United States [18].

Previous research has indicated that legume consumers are more likely to be Hispanic and with a greater education level [19]. While another study found that consumption of chickpeas was highest among non-Hispanic Asians and those with a greater education level [20], an understanding of sociodemographic patterns in overall consumption of lentils/dried peas is lacking. Examining lentil and dried pea consumption patterns over time and across socio-demographic groups would provide an understanding of the degree of acceptance of these foods in the American diet. Additionally, investigation of the culinary use of lentils and dried peas would inform opportunities to increase intake of these foods. The objective of this study is to examine the trends in the prevalence of lentils/dried peas consumption in the US using National Health and Nutrition Examination Survey (NHANES) data from 2003–2004 through 2017–2018. An additional objective is to examine the individual foods and dishes in which lentils and dried peas are consumed, and identify the meal context and the retail and other sources of lentils and dried peas among consumers using NHANES 2017–2018.

## 2. Materials and Methods

### 2.1. Study Population

The NHANES is a cross-sectional survey that collects data on health and nutritional status of the noninstitutionalized civilian U.S. population [21]. The NHANES sample is nationally representative and is selected via complex, stratified, multistage probability cluster-sampling. The current study utilized 2-year NHANES cycles from 2003–2004 through 2017–2018 for the trend analyses. The trend analyses included adults (aged 18 years or older) and children (aged 3–17 years) with data on dietary intake. The resulting sample sizes for adults are 4986, 5060, 5690, 6052, 5076, 5356, 5266 and 4983 in NHANES 2003–2004, 2005–2006, 2007–2008, 2009–2010, 2011–2012, 2013–2014, 2015–2016 and 2017–2018, respectively. Corresponding sample sizes in children are 3025, 3182, 2553, 2699, 2583, 2504, 2432 and 1971. The NHANES protocol has been approved by the National Center for Health Statistics’ Research Ethics Review Board [22] and written informed consent was obtained from participants. Since publicly available, de-identified datasets were used in the current research, the study is considered as ‘not human subjects research’ and did not require IRB approval.

### 2.2. Identifying Consumers of Lentils and Dried Peas

To assess intake of lentils/dried peas, the study used information on foods and beverages consumed during the preceding 24-h period, collected via 24-h recalls [23]. NHANES uses up to two 24-h recalls to collect dietary information from participants; for participants who completed only one 24-h recall, dietary data from one recall was used. While the first 24-h recall is collected in the Mobile Examination Center, the second recall is collected via phone 3–10 days later. For children aged less than 6 years, 24-h recalls were reported by the person most knowledgeable about the child’s dietary intake [24], whereas proxies assisted dietary recall collection for children aged 6–11 years. Dietary intakes are self-reported by participants aged ≥12 years.

Publicly-available individual food files [25,26] and food code description files [27] were used to assess the prevalence of lentils/dried peas consumption and the eating context. Food codes corresponding to lentils/dried peas and foods containing lentils/dried peas were identified from the food code description file. They were then compared against the corresponding legume intake, obtained from the Food Patterns Equivalents Database, to ensure that the extracted food codes are classified as legumes [28]. These food codes were used to identify NHANES participants who consumed lentils/dried peas on at least one day of the diet recall. Those reporting intake of lentils/dried peas for at least one day were classified as consumers, whereas others were categorized as non-consumers. In addition, the eating occasion (e.g., lunch, dinner) and retail/other sources of lentils/dried peas were obtained from the individual food files.

### 2.3. Sociodemographic and Dietary Characteristics

Sociodemographic characteristics included for the analyses were participants’ age, sex, race/ethnicity, education level, language spoken at home (for non-Hispanic Asian and Hispanic adults) and family income to poverty ratio. Dietary characteristics included were diet quality, as assessed using the Healthy Eating Index-2015 (HEI-2015), and protein intake. The HEI-2015 measures adherence of dietary intake to the 2015–2020 Dietary Guidelines for Americans (DGA) [29]. The HEI-2015 score, with a maximum possible value of 100, is a summation of scores that indicate conformance of intake of nine adequacy components (total fruits, whole fruits, total vegetables, greens and beans, whole grains, dairy, total protein foods, seafood and plant proteins, and fatty acids) and four moderation components (refined grains, sodium, added sugars and saturated fat) dietary components to recommendations, with higher scores reflective of greater adherence to guidelines. Additionally, those who did not report any consumption of meat, poultry/eggs and seafood in their dietary recalls were classified as vegetarians.

### 2.4. Statistical Analyses

Normality of distribution was checked by skewness and kurtosis. Absolute skewness or kurtosis values of >2 or >7, respectively, were considered to indicate substantial departure from normality [30]. All continuous variables were normally distributed. Linear regression and chi-square tests examined demographic and dietary differences between consumers and non-consumers. Multivariable linear regression models examined time trends in lentils and dried peas consumption from 2003–2004 to 2017–2018 in adults (aged 18 years or older) and children (aged 3–17 years). Analyses involving children were adjusted for age, sex, race/ethnicity, and family income to poverty ratio; analyses involving adults were additionally adjusted for education level. Analyses accounted for the complex survey design of NHANES.

Among lentils/dried peas consumers, chi-square tests were used to indicate the distribution in the proportion of eating occasions, individual foods, and retail and other sources of lentils/dried peas. All analyses were conducted in the SAS (version 9.4; SAS Institute, Inc., Cary, NC, USA) software.

## 3. Results

### 3.1. Trends in Prevalence of Lentils/Dried Peas Consumption in Adults and Children

**Figure 1:**
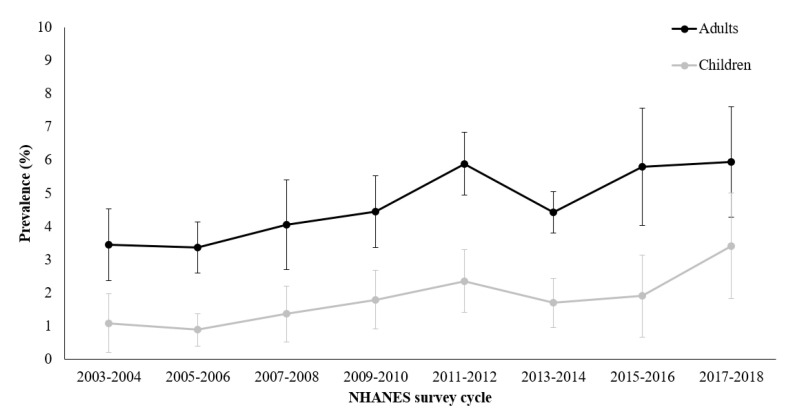
Trends in prevalence (95% confidence interval) of lentils/dried peas consumption among adults and children from NHANES 2003–2004 through 2017–2018.

In adults, the prevalence of lentils/dried peas consumption increased from 3.5% in NHANES 2003–2004 to 6.0% in NHANES 2017–2018 (Figure 1). In children, the prevalence of lentils/dried peas consumption increased from 1.1% in NHANES 2003–2004 to 3.4% in NHANES 2017–2018. The increasing prevalence of lentils/dried peas consumption observed over the study period was statistically significant in both adults (*p* value for trend = 0.02) and children (*p* value for trend = 0.003).

### 3.2. Demographic Characteristics of Lentils/Dried Peas Consumers and Non-Consumers in NHANES 2017–2018

Among adults, a significantly greater proportion of non-Hispanic Asians, those with a college degree or above and vegetarians (as imputed by absence of meat, poultry, or fish from diet recall data) were classified as consumers of lentils/dried peas (Table 1). Among Hispanic and non-Hispanic Asian adults, the language spoken at home (English versus non-English languages) was not significantly different between consumers and non-consumers. Lentils/dried peas consumers had a significantly higher income to poverty ratio and HEI-2015 score compared to non-consumers. Protein intake was not significantly different between consumers and non-consumers. Similar demographic differences between consumers and non-consumers were observed in children.

### 3.3. Eating Context of Lentils and Dried Peas in NHANES 2017–2018

Lentil curry and hummus were the most consumed individual dishes in lentils and dried peas categories, accounting for 36.5% and 52.1% of occurences respectively (Table 2). Lentils and dried peas were commonly consumed during lunch and dinner, accounting for 87.4% and 63.4% of occurences, respectively (Table 3). Consumers of lentils and dried peas prrimarly obtained these foods from grocery stores and supermarkets, which accounted for 84.8% lentil servings and 73.6% of dried pea servings (Table 4). Restaurants and fast food outlets were the second most common source of both foods.

## 4. Discussion

In this nationally representative study, we found a significantly increasing trend in consumption of lentils/dried peas from NHANES 2003–2004 through 2017–2018. Despite the increasing trend, lentils/dried peas consumption was still low in NHANES 2017–2018 with a prevalence of about 6% in adults and 3% in children. Further, the increasing trend in lentils/dried peas consumption was not consistently observed over the study period, such that a decline in prevalence was observed from NHANES 2011–2012 to NHANES 2013–2014. Although the reason for this decline is unclear, this result is comparable to another study noting a decrease in legume consumption from NHANES 2011–2012 to 2013–2014 [18]. Significant differences in race/ethnicity were observed between consumers and non-consumers, such that a greater proportion of consumers were non-Hispanic Asians compared to non-consumers. Previous studies have indicated a higher prevalence of legume intake among Hispanic individuals [31,32], especially those with lower acculturation [33]; in contrast, we found that the proportion of consumers who were Hispanic was lower than that of non-consumers. Additionally, lentils/dried peas intake was not significantly different by language spoken at home, a commonly used a proxy for acculturation [34,35], among Hispanic adults. The focus on lentils and dried peas in the current study may explain the difference in findings from previous research, and suggest that dried beans (e.g., pinto and black turtle beans) could be the predominant pulse consumed by Hispanic individuals [36]. Although pulses are considered to be an inexpensive source of protein [37], we found that consumers had a significantly higher income to poverty ratio compared to non-consumers. This finding is comparable to a previous study based on NHANES data indicating a greater prevalence of chickpea consumption among those with higher incomes [20]. However, that previous study also found that consumption of other sources of legumes (i.e., not including chickpeas) did not vary by income [20]; a discrepancy that could be explained by the focus on lentils/dried peas in the current study. Nevertheless, since low-income individuals may face financial constraints in purchasing of foods [38] and are more likely to consume lower quality diets, marked by lower intake of vegetables [39], incorporation of varied sources of legumes, including lentils/dried peas, could help improve their diet quality while still being economically effective.

In the current study, lentils/dried peas consumers had a significantly higher dietary quality, based on the HEI-2015, compared to non-consumers. While consumption of legumes may improve diet quality by helping meet the dietary recommendations for vegetables [17], inclusion of lentils/dried peas alone may not fully explain the observed differences in diet quality between consumers and non-consumers. Since a greater proportion of lentils/dried peas consumers were classified as vegetarians, it is possible that consumption of an overall healthful diet intake, marked by greater plant-based foods such as fruits and vegetables [40], could additionally explain the observed differences in diet quality. We did not find any differences in protein intake between lentils/dried peas consumers and non-consumers.

An understanding of individual foods and dishes that contribute to lentils/dried peas intake is vital for efforts that target to improve acceptance of these foods. In the current study, lentil curry and hummus were the most commonly consumed individual foods within the lentils and dried peas category, respectively. Further, hummus constituted about half of the individual foods within the dried peas category; a finding that could reflect the increasing trend in the consumption of hummus over the years [20]. The predominant share of lentils/dried peas was obtained from grocery stores/supermarkets, whereas convenience stores accounted for less than 3% of the source of acquisition. With efforts to improve availability of healthful foods in convenience stores [41,42], future strategies could focus on opportunities to increase legume availability and acceptance of these foods among customers who rely on convenience stores for food purchases.

A limitation of this study is that the cross-sectional nature of the study design. This study used 24-h recalls to assess dietary intake, a methodology that may not capture habitual intake and could be subject to biases arising from self-reports [43]. Given the low prevalence of lentils/dried peas consumption, 24-h recalls could also have missed some individuals who infrequently consume lentils/dried peas. Strengths of the current study include the use of a large, nationally representative sample and multiple-pass method in 24-h recalls [23], where follow-up questions were asked by interviewers to improve the accuracy of dietary assessment.

## 5. Conclusions

The inclusion of peas and lentils could be an affordable way to improve dietary quality yet the prevalence of consumption was low among adults and children participating in NHANES 2017–2018. Demographic differences between consumers and non-consumers of lentils/dried peas imply greater acceptance of these foods among non-Hispanic Asians and those from a higher socioeconomic status. Hummus represents a commonly consumed food containing dried peas, and grocery store/supermarket represents a major source of acquisition of lentils/dried peas. Although there are signs of rising acceptance of lentils and dried peas, additional studies investigating the barriers to consumption of these foods could further identify opportunities for incorporating these foods in the diets of Americans.

## Figures and Tables

**Table 1:** Demographic and dietary characteristics ^a^ of US adults (*n* = 4983) and children (*n* = 1971) by consumption of lentils/dried peas: National Health and Nutrition Examination Survey 2017–2018.

Characteristics	Adults, Aged 18 Years or Older		Children, Aged 3–17 Years	
	Non-Consumers (*n* = 4690)	Consumers (*n* = 293)	Non-Consumers (*n* = 1913)	Consumers (*n* = 58)
Age	46.9 ± 0.7	47.0 ± 1.7	10.2 ± 0.2	10.0 ± 0.9
Sex				
Male	48.8	41.1	52.0	39.7
Female	51.2	58.9	48.0	60.3
Race/ethnicity				
Mexican American	9.8	6.2 ***	17.8	9.4 ***
Other Hispanic	7.2	5.2	7.2	7.6
Non-Hispanic White	61.3	61.3	49.8	51.7
Non-Hispanic Black	11.7	4.0	12.4	5.0
Non-Hispanic Asian	5.2	20.7	4.3	22.3
Other	4.7	2.6	8.5	4.0
Education level				
<9th grade	3.6	2.5 ***		
9–11 grade	7.2	2.7		
High school graduate	28.9	11.2		
Some college/associate degree	31.8	18.6		
College graduate or above	28.6	65.0		
Language spoken at home (Hispanics)				
Only Spanish	27.1	22.0		
More Spanish than English	16.3	26.0		
Both equally	15.0	14.9		
More English than Spanish	16.1	13.6		
Only English	25.5	23.5		
Language spoken at home (Asians)				
Only Non-English language	39.5	48.4		
More Non-English than English	10.9	9.7		
Both equally	11.6	15.1		
More English than Non-English	8.6	9.3		
Only English	29.4	17.6		
Healthy Eating Index 2015 total score	46.9 ± 0.7	60.2 ± 1.6 ***	46.8 ± 0.5	58.8 ± 2.5 ***
Protein intake, % kilocalories	16.1 ± 0.1	16.7 ± 0.8	14.4 ± 0.2	14.8 ± 1.1
Vegetarian, based on dietary recall data				
No	98.8	92.2 ***	98.2	94.4 *
Yes	1.2	7.8	1.8	5.6
Income to poverty ratio	3.0 ± 0.1	3.7 ± 0.2 ***	2.4 ± 0.1	3.7 ± 0.3 ***

* *p* < 0.05; *** *p* < 0.001. ^a^ Data are represented as mean ± standard error of mean or %.

**Table 2:** Distribution of individual foods among lentils and dried peas consumers, National Health and Nutrition Examination Survey 2017–2018.

Food Source	Proportion of Occurrence (%)
Lentils	
Lentil curry (with or without rice)	36.5
Lentil soup	33.5
Lentils, from dried (with or without fat added)	21.0
Lentils, not further specified	5.1
White rice with lentils (with or without fat added)	3.0
Lentils, from canned	1.0
Peas	
Hummus, plain	27.2
Hummus, flavored	24.9
Chickpeas, not further specified	14.3
Blackeyed peas (canned or frozen)	9.7
Chickpeas, from dried (with or without fat)	5.5
Chickpeas, from canned (with or without fat)	5.1
Blackeyed peas, from dried	4.2
Split pea soup	3.7
Blackeyed peas, not further specified	2.3
Split pea and ham soup	2.3
Garbanzo bean or chickpea soup, home recipe, canned or ready-to-serve	0.9

**Table 3:** Distribution of eating occasions ^a^ among lentils and dried peas consumers, National Health and Nutrition Examination Survey 2017–2018.

Eating Occasion	Proportion of Occurrence (%)
Lentils	
Breakfast	4.1
Lunch/almuerzo	38.6
Dinner/cena	48.8
Supper	2.4
Snack/comida	6.1
Peas	
Breakfast	3.2
Lunch/almuerzo	33.3
Dinner/cena	30.1
Supper	5.6
Brunch	2.3
Snack/comida/entre comida/botana	25.5

^a^ The eating occasion ‘infant feeding’ was excluded from analyses.

**Table 4:** Distribution of retail and other sources of lentils and dried peas among consumers, National Health and Nutrition Examination Survey 2017–2018.

Eating Occasion	Proportion of Occurrence (%)
Lentils	
Grocery store/supermarket	84.8
Restaurant/fast food joint	9.5
Convenience store	2.7
Cafeteria (school or outside school)/child or adult care center	1.0
From someone else	2.0
Peas	
Grocery store/supermarket	73.6
Restaurant/fast food joint	11.6
Cafeteria (school or outside school)	6.5
From someone else	6.0
Convenience store	1.4
Grown by self/others	0.5
Common snack tray	0.5

## Data Availability

Data available on request.
